# Supplementary material for: Asymptomatic infection and antibody prevalence to co-occurring avian influenza viruses vary substantially between sympatric seabird species following H5N1 outbreaks
Source: Sci Rep. 2025 Jan 9;15:1435. doi: 10.1038/s41598-025-85152-6 (PMC11718005; doi:10.1038/s41598-025-85152-6)
Supplement: Supplementary file 1 — Supplementary Information. [file 41598_2025_85152_MOESM1_ESM.docx]

**Asymptomatic infection and antibody prevalence to co-occurring avian influenza viruses vary substantially between sympatric seabird species following H5N1 outbreaks**

Fiona Greco^1*^, Hannah M. Ravenswater^1^, Francisco Ruiz-Raya^1^, Chiara D’Avino^1^, Mark A. Newell^2^, Josie Hewitt^2^, Erin Taylor^2^, Ella Benninghaus^2^, Francis Daunt^2^, Gidona Goodman^3^, David Steel^4^, Jenny Park^4^, Emma Philip^4^, Saumya S. Thomas^5^, Marek J. Slomka^5^, Marco Falchieri^5^, Scott M. Reid^5^, Joe James^5^, Ashley C. Banyard^5^, Sarah J. Burthe^2†^ and Emma J.A. Cunningham^1†^

^1^Institute of Ecology and Evolution, School of Biological Sciences, University of Edinburgh, Ashworth Laboratories, King’s Buildings, Charlotte Auerbach Road, Edinburgh, EH9 3FL, UK
^2^UK Centre for Ecology & Hydrology, Bush Estate, Penicuik, Scotland, EH26 0QB, UK

^3^Royal (Dick) School of Veterinary Studies and the Roslin Institute, University of Edinburgh, Easter Bush Campus, Midlothian EH26 9RG

^4^NatureScot, Great Glen House, Inverness, IV3 8NW, UK  ^5^Influenza and Avian Virology team, Department of Virology, Animal and Plant Health Agency, Weybridge, Addlestone, Surrey, KT15 3NB, UK

**†** Shared last authorship

* Fiona.Greco@ed.ac.uk

**Supplementary information**

**Mortality events – summer 2023**

Two kittiwake carcasses swabbed on 26^th^ June 2023 tested positive for AI viral RNA. A total of 871 kittiwake deaths were subsequently reported as suspected H5N1, including 190 fledglings and 681 sub-adults/adults. These deaths were predominantly concentrated in a single geographical location around a freshwater loch. Mortality of unknown cause was also recorded in 30 individuals of other species, namely herring gull (n=6), lesser-black backed gull (n=3), puffin (n=17) and eider (n=4) (*NatureScot, unpublished data*). These latter records of mortality across the season do not appear atypical in level in for these species.

***Supplementary Table S1*:** *Glossary box of key avian influenza terms*

| **Term(s)** | **Definition** |
| --- | --- |
| Avian influenza viruses (AIVs) | Influenza A viruses of the *Orthomyxoviridae* family, infecting both domestic and wild avian species, and naturally circulating in wild aquatic birds. Also associated with sporadic infection of mammals, including humans^1^ |
| AIV subtypes | Avian influenza viruses form multiple serological subtypes, characterised by surface glycoproteins haemagglutinin (H1-H16) and neuraminidase (N1-N9)^2^ |
| A/goose/Guangdong/1/1996 (Gs/GD) | H5 viral lineage first described in a domestic goose in Guangdong, China in 1996. Following spill-back into wild populations, this lineage has evolved and spread across multiple continents, with disease in domestic and wild birds, and mammals^3^ |
| AB and BB genotypes | Two of several HPAI H5Nx genotypes identified in Europe between July 2020 and August 2022  BB genotype refers to a reassortant genotype emerging in 2022, associated with gull-adaptation and mass mortality events in gull species^4^ |
| Clade 2.3.4.4b | One of several phylogenetically distinct AIV sub-clades derived from evolution and reassortment of A/goose/Guangdong/1/1996 (Gs/GD) lineage^3,5^ |
| High pathogenic avian influenza (HPAI) | Category of AIV classified as such based on pathogenicity and mortality in chickens, and sequence of HAO cleavage site amino acid present^6^. Associated with severe clinical signs and possible high mortality rates^6^. |
| Low pathogenic avian influenza (LPAI) | Classified based on pathogenicity of virus in poultry; LPAI associated with little or no clinical signs of disease^6^. |

***Supplementary Table S2*:** *Glossary box of general terms*

| **Term(s)** | **Definition** |
| --- | --- |
| Asymptomatic | A case with no obvious outward signs of infection  NB. some fields prefer the use of sub-clinical for animals in veterinary settings, while others use the term interchangeably with asymptomatic |
| ELISA  (Enzyme-linked immunosorbent assay) | Immunological assay used here for the detection of host antibodies against  Influenza A virus in collected plasma samples. |
| Exposure | Contact of host with infectious pathogen^7^ |
| Haemagglutination Inhibition assay | Immunological assay used here to detect host antibodies to AIV, and used to **subtype** these antibodies as specific to a particular AIV subtype. Here, we first test for antibodies specific to the H5 HA protein, providing evidence of host infection with this specific AIV subtype. Where birds test positive for AIV but not H5 we further test for H13 and H16, commonly considered to be associated with certain seabird taxa |
| Recombination | The exchange or switching of genetic material when two viral genomes co-infect the same cell^8^, producing novel viral strains. |
| Reassortment | A version of recombination specific to segmented viruses, permitting the exchange of complete parts of the genome between viral co-pathogens in the cells of the same host^5^. Potential to form novel strains of increased virulence and altered host adaptation^9^, which may facilitate rapid host switching. |
| Resistance | Host strategy to limit within-host survival of pathogen or reduce likelihood of infection, reducing pathogen prevalence in a host population^10^. |
| rRT-PCR  (Real-time Reverse- Transcription  Polymerase Chain Reaction) | Here, used as a molecular method for the detection and quantification of AIV RNA^2^. Different assays use differing target regions of the viral genome to amplify and detect AIV, with subtyping ability dependant on target region |
| Susceptibility | Vulnerability of host to infection. Reflects extent of successful infection following exposure to pathogen^7^. |
| Tolerance | Limitation of the negative fitness effect of parasites by a host^10^ |

***Supplementary Table S3:***  *Predominant species assemblage on the Isle of May NNR and relevant UK Bird of Conservation Concern status. Where available, breeding population estimates for each species on the IOM are presented for the year prior to sampling (2022): AON = apparently occupied nests, AOS = apparently occupied sites, AOB = apparently occupied burrows. Where data is not available for 2022, the previous census data is given – kittiwakes (2021) and puffins (2017). With the exception of eiders where the figure for breeding males is unknown, these values represent breeding pairs and the breeding population of individuals will be double these figures. Note that non-breeders present are not included in these estimates. Also noted is the UK breeding population as a percentage of the international population. Finally, the percentage change in breeding population size of each species in the designated Forth Islands Special Protection Area between the seabird population census’ Seabird 2000 (1998-2002) and Seabirds Count (2015-2021) is given. These population changes pre-date the HPAI H5N1 clade 2.3.4.4b epizootic*, *with some species already showing clear declines prior to the emergence of HPAIV.*

| **Species** | **Isle of May Species Estimates 2022^11,12^** | **UK Birds of Conservation Concern 5^13,14^** | **UK population as % of international population^15^** | **Forth Island SPA % breeding population change^16^** |
| --- | --- | --- | --- | --- |
| Puffin  (*Fratercula arctica*) | 39,200 AOB  (*2017 census*) | Red | 9.6% | -40% |
| Black-legged kittiwake (*Rissa tridactyla*) | 5193 AON  (*2021 census*) | Red | 8% | -22% |
| Herring gull  (*Larus argentatus*) | 5168 AON | Red | 12.1% | -14% |
| Arctic tern  (*Sterna paradisaea*) | 578 AON | Red | 3.1% | -8% |
| Great black-backed gull (*Larus marinus*) | 116 AON | Red | 9.6% | NA |
| European shag  (*Gulosus aristotelis*) | 467 AON | Amber | 34.1% | -30% |
| Lesser black-backed gull (*Larus fuscus*) | 1739 AON | Amber | 38.4% | -31% |
| Common guillemot (*Uria aalge*) | 17,318 AOS | Amber | 12.9% | -27% |
| Northern fulmar (*Fulmarus glacialis*) | 321 AON | Amber | 8% | NA |
| Razorbill  (*Alca torda*) | 4381 AOS | Amber | 20.2% | +23% |
| Common eider (Somateria mollissima) | 715 nesting females | Amber | NA | NA |

***Supplementary Table S4*:** *Number of total positive (Cq ≤36.0) or borderline (Cq >36.0, <40.0) swab samples per number of individuals for each species. M-gene assay refers to the detection of nucleic acid from all sixteen generic HA AIV subtypes, while H5HP assay is specific for nucleic acid of HPAI H5Nx pathotype. Species prevalence for positive and borderline results is given in brackets, unless zero detection*. *Both positive and borderline results are displayed in* ***bold****. Range of infected individuals gives a minimum and maximum percentage of positive viral detection of AIV (all subtypes) per samples for each species. The minimum value indicates confirmed positive individuals only, while maximum value combines positive and borderline individuals. Note that, one individual puffin tested borderline across two assays, so the total number of borderlines plus positive results in puffins is n=6. Our estimate of positive individuals across the entire set sampled ranged from 1.6-4.5% (5/309 to 14/309)*.

| **Species** | **M-gene** | | **H5 HP** | | **N1** | | **Range of infected individuals** |
| --- | --- | --- | --- | --- | --- | --- | --- |
|  | **Positive** | **Borderline** | **Positive** | **Borderline** | **Positive** | **Borderline** |  |
| Shag | 0/98 | 0/98 | 0/98 | 0/98 | NA | NA | NA |
| Kittiwake | 0/74 | 0/74 | 0/74 | 0/74 | NA | NA | NA |
| Puffin | **1/45 (2.2%)** | **3/45 (6.7%)** | 0/45 | **3/45 (6.7%)** | 0/3 | **1/3** | 2.3% (1/45) –  13.3% (6/45) |
| Guillemot | **3/44**  **(6.8%)** | **4/44**  **(9.1%)** | 0/44 | 0/44 | **1/7** | **2/7** | 6.8% (3/44) – 15.9% (7/44) |
| Razorbill | 0/48 | 0/48 | **1/48 (2.1%)** | 0/48 | 0/1 | 0/1 | 2.1% |

***Supplementary Table S5:*** *Results breakdown across assay type in individuals reported positive (Cq ≤36.0) or borderline (given here, Cq 36.01 – 36.9) for viral nucleic acid via rRT-PCR (positives in* ***bold****). Cq value or negative result given, NA = assay not performed. Swab type CL= detection on cloacal swab, CH= detection on choanal swab. An indication of the final subtyping achieved for each positive sample is given: HxNx = AIV of unknown subtype; H5Nx = high pathogenicity H5, unconfirmed neuraminidase subtype; HxN1 = confirmed N1, unconfirmed haemagglutinin subtype. Associated competitive ELISA assay (H1-H16 and H5 specific) and haemagglutination inhibition (HI) results also given for each individual.*

| **Species** | **Sample date** | **Swab type** | **Assay type** | | | **Indicated subtype of positive samples** | **ELISA**  **(H1-H16)** | **ELISA**  **(H5)** | **H5 HI** |
| --- | --- | --- | --- | --- | --- | --- | --- | --- | --- |
|  |  |  | **M-gene** | **H5 HP** | **N1** |  |  |  |  |
| **Puffin** | **06/07/2023** | OP | **35.64** | No Cq | No Cq | HxNx | Negative | Not tested | Negative |
|  |  | C | No Cq | No Cq | NA |  |  |  |  |
| **Guillemot** | **21/06/2023** | OP | No Cq | No Cq | NA | HxNx | **Positive** | **Positive** | Negative |
|  |  | C | **34.38** | No Cq | 36.43 |  |  |  |  |
| **Guillemot** | **21/06/2023** | OP | **35.85** | No Cq | No Cq | HxNx | Negative | Not tested | Negative |
|  |  | C | No Cq | No Cq | NA |  |  |  |  |
| **Guillemot** | **22/06/2023** | OP | No Cq | No Cq | NA | HxN1 | **Positive** | **Positive** | Negative/ |
|  |  | C | **34.28** | No Cq | **35.02** |  |  |  | Weak reactor |
| **Razorbill** | **17/06/2023** | OP | No Cq | **34.97** | No Cq | HP H5Nx | Negative | Not tested | Negative |
|  |  | C | No Cq | No Cq | NA |  |  |  |  |
| Puffin | 11/06/2023 | OP | No Cq | No Cq | NA |  | Not tested | Not tested | Negative |
|  |  | C | 36.36 | 36.55 | 38.25 |  |  |  |  |
| Guillemot | 21/06/2023 | OP | 36.62 | No Cq | No Cq |  | Negative | Not tested | Negative |
|  |  | C | No Cq | No Cq | NA |  |  |  |  |
| Guillemot | 21/06/2023 | OP | 36.4 | No Cq | No Cq |  | Positive | Negative | Negative |
|  |  | C | No Cq | No Cq | NA |  |  |  |  |
| Guillemot | 21/06/2023 | OP | No Cq | No Cq | NA |  | Negative | Negative | Negative |
|  |  | C | 36.89 | No Cq | 36.43 |  |  |  |  |
| Guillemot | 21/06/2023 | OP | 36.75 | No Cq | No Cq |  | Positive | Negative | Negative |
|  |  | C | No Cq | No Cq | NA |  |  |  |  |
| Puffin | 06/07/2023 | OP | 36.91 | No Cq | NA |  | Positive | Negative | Negative |
|  |  | C | No Cq | No Cq | NA |  |  |  |  |
| Puffin | 29/06/2023 | OP | No Cq | 37.5 | NA |  | Negative | Not tested | Negative |
|  |  | C | No Cq | 39.09 | NA |  |  |  |  |
| Puffin | 29/06/2023 | OP | No Cq | No Cq | NA |  | Negative | Not tested | Negative |
|  |  | C | 38.32 | No Cq | NA |  |  |  |  |
| Puffin | 06/07/2023 | OP | No Cq | No Cq | NA |  | Negative | Not tested | Negative |
|  |  | C | No Cq | 38.68 | NA |  |  |  |  |

**References**

1 Olsen, B. *et al.* Global Patterns of Influenza A Virus in Wild Birds *Science* **312** (2006).

2 Spackman, E. in *Animal Influenza Virus: Methods and Protocols* *Methods in Molecular Biology* (ed E. Spackman) 83-92 (Humana Press, 2020).

3 Lee, D. H., Bertran, K., Kwon, J. H. & Swayne, D. E. Evolution, global spread, and pathogenicity of highly pathogenic avian influenza H5Nx clade 2.3.4.4. *J Vet Sci* **18**, 269-280 (2017). <https://doi.org:10.4142/jvs.2017.18.S1.269>

4 Fusaro, A. *et al.* High pathogenic avian influenza A(H5) viruses of clade 2.3.4.4b in Europe-Why trends of virus evolution are more difficult to predict. *Virus Evol* **10**, veae027 (2024). <https://doi.org:10.1093/ve/veae027>

5 Lycett, S. J., Duchatel, F. & Digard, P. A brief history of bird flu. *Philos Trans R Soc Lond B Biol Sci* **374**, 20180257 (2019). <https://doi.org:10.1098/rstb.2018.0257>

6 WOAH. *Chapter 3.3.4, Avian Influenza (Including Infection with High Pathogenicity Avian Influenza Viruses)*, <<https://www.woah.org/fileadmin/Home/eng/Health_standards/tahm/3.03.04_AI.pdf>> (2023).

7 Sweeny, A. R. & Albery, G. F. Exposure and susceptibility: The Twin Pillars of infection. *Functional Ecology* **36**, 1713-1726 (2022). <https://doi.org:10.1111/1365-2435.14065>

8 Shao, W., Li, X., Goraya, M. U., Wang, S. & Chen, J. L. Evolution of Influenza A Virus by Mutation and Re-Assortment. *Int J Mol Sci* **18** (2017). <https://doi.org:10.3390/ijms18081650>

9 Kandeil, A. *et al.* Rapid evolution of A(H5N1) influenza viruses after intercontinental spread to North America. *Nat Commun* **14**, 3082 (2023). <https://doi.org:10.1038/s41467-023-38415-7>

10 Kutzer, M. A. & Armitage, S. A. Maximising fitness in the face of parasites: a review of host tolerance. *Zoology (Jena)* **119**, 281-289 (2016). <https://doi.org:10.1016/j.zool.2016.05.011>

11 Outram, B. & Steel, D. *Isle of May NNR - Annual Report 2021* <<https://www.nature.scot/doc/isle-may-nnr-annual-report-2021#1.4.1+Puffin>> (2023).

12 Steel, D. & Greetham, H. *Isle of May National Nature Reserve Annual Report 2022*, <<https://www.nature.scot/doc/isle-may-national-nature-reserve-annual-report-2022#2+Highly+Pathogenic+Avian+Influenza+(HPAI>)> (2023).

13 Stanbury, A. J. *et al.* The status of our bird populations: the fifth Birds of Conservation Concern in the United Kingdom, Channel Islands and Isle of Man and second IUCN Red List assessment of extinction risk for Great Britain. *British Birds* **114** (2021).

14 Stanbury, A. J. *et al.* The status of the UK’s breeding seabirds: an addendum to the fifth Birds of Conservation Concern in the United Kingdom, Channel Islands and Isle of Man and second IUCN Red List assessment of extinction risk for Great Britain. *British Birds* **117**, 471-487 (2024).

15 JNCC. Seabird Population Trends and Causes of Change: 1986–2019 Report. (Peterborough, 2021).

16 Burnell, D. *et al.* *Seabirds Count: a census of breeding seabirds in Britain and Ireland (2015-2021)*, 2023).
